# MHF-Net: Multi-Modal Residual Fusion Object Detection Network Based on High-Frequency Gated Convolution

**DOI:** 10.3390/s26185816

**Published:** 2026-09-14

**Authors:** Bailin Chen, Bo Qian

**Affiliations:** School of Information Science and Engineering, Shenyang Ligong University, Shenyang 100159, China; 2503620512@stu.sylu.edu.cn

**Keywords:** autonomous driving, multi-modal, high-frequency gated convolution, dual-path attention mechanism, spatial-channel downsampling module

## Abstract

This paper aims to address image degradation caused by adverse weather conditions and partial occlusion in autonomous driving scenarios. The network adopts a dual-branch architecture, which utilizes a residual fusion block (ResFuse) to achieve the deep fusion of cross-modal features. A dual-path attention mechanism (DualAtt) and an integrated high-frequency attention module (HFAtt) were designed to capture key information in feature maps. Additionally, this study designs a spatial-channel downsampling module (SCDown). This module implements spatial-to-channel image downsampling. Experimental results demonstrate that the proposed network achieves a detection speed of 61 frames per second (FPS) and precision (P) of up to 87.4% on the M3FD dataset. Comparative experimental results show that MHF-Net achieves 84.3% in the mAP_50_ evaluation metric. Compared to the baseline model, the number of parameters was reduced by 95% and the computational complexity was reduced by 88%. Compared with other mainstream object detection algorithms, MHF-Net strikes a favorable balance regarding performance in autonomous driving scenarios.

## 1. Introduction

Object detection is a core technology in the field of computer vision [1] that achieves the automatic recognition and localization of objects, providing a solid foundation for subsequent tasks such as decision analysis and behavior understanding. Object detection is widely applied in multiple fields, such as obstacle detection in autonomous driving, defect detection in intelligent manufacturing, and target detection in aerial drones, and has received extensive attention across both academia and industry. However, the application of object detection algorithms in autonomous driving scenarios is limited by image blurring issues caused by severe weather in practical detection scenes, which severely affect the robustness and accuracy of detection [2]. Existing single-modal object detection algorithms such as the DETR series [3] and YOLO series [4,5] struggle to effectively handle the aforementioned complex scenarios because they lack the capability to comprehensively utilize multi-modal information. Therefore, multi-modal object detection fusion and feature processing based on RGB and thermal infrared images have become a research hotspot, attracting increasing attention from researchers.

In recent years, object detection algorithms have been mainly divided into two categories: single-modal (RGB, IR, ID) and multi-modal (RGB + IR, RGB + ID, RGB + Label). Single-modal object detection algorithms are mainly categorized into two-stage, one-stage, and Transformer-based detection algorithms, constituting the mainstream research directions in this field. Two-stage detection algorithms (e.g., Sparse R-CNN [6], Dynamic R-CNN [7]) achieve high-precision detection through a two-step process of “region proposal generation and classification”, which constitutes the core detection pipeline. The core of this approach lies in constructing multi-scale anchor boxes, which are then fine-tuned based on the intersection over union (IoU) and cross-entropy loss (CE loss). One-stage detection algorithms (e.g., the YOLO series [8,9]) abandon region proposal generation, directly transforming detection into an end-to-end regression problem. These algorithms output predictions based on multi-scale feature maps and pre-defined anchor boxes, which are then filtered by non-maximum suppression (NMS) to select optimal bounding boxes for efficient inference. Transformer-based detection algorithms (e.g., DETR [10], DN-DETR [11]) adopt a truly end-to-end minimalist architecture, but they face the challenges of slow convergence and high computational costs. Their core mechanism extracts 2D feature maps from input images, which are then combined with positional encoding to directly output detection results. Single-modal detection methods are structurally simple and computationally efficient, but they suffer from limitations due to insufficient information in complex scenarios.

Multi-modal object detection algorithms primarily rely on three strategies: early fusion, mid-level fusion, and late fusion. Early fusion (TarDAL [12], Beyond RG [13]) leverages neural networks to fuse multi-modal images into a single high-quality image prior to detection. Mid-level fusion (MBNet [14], CORE-Net [15]) utilizes cross-modal attention mechanisms and Transformer architectures to address issues of feature misalignment and modality imbalance. Late fusion (e.g., MSDNN [16], MSCoTDet [17]) concatenates feature vectors from the fully connected layers of each modality, leaving the joint decision to the final classifier. By fusing information from multiple sensors, multi-modal methods can effectively compensate for the limitations of a single modality and enhance the detection robustness.

However, existing multi-modal fusion strategies still have many limitations [18]. For example, early fusion leads to modality feature loss and noise superposition. Late fusion results in doubled memory and computational costs, which makes it difficult to achieve real-time detection on edge devices. Intermediate fusion alleviates spatial misalignment through deep feature downsampling; however, it still faces the challenge of low efficiency in cross-modal information interaction. In addition, traditional methods [19,20,21,22] that borrow effective information from other modalities improve performance by stacking a large number of modules, which allows performance gains but at the cost of a significantly increased computational burden.

To address the above limitations, this paper proposes a multi-modal residual fusion object detection network based on high-frequency gated convolution (MHF-Net), which aims to improve the multi-modal object detection performance while reducing the computational resource consumption in RGB-IR autonomous driving scenarios.

The main contributions of this paper are summarized as follows:

- A residual fusion block (ResFuse) is designed, adopting a dual-branch architecture to achieve the deep fusion of cross-modal features. This module exploits the complementary relationship between RGB and thermal modalities, enhancing feature responses in target regions while suppressing feature representations in background regions. This provides rich feature information for long-range modeling.
- A dual-path attention mechanism (DualAtt) and a high-frequency attention module (HFAtt) are designed. These modules process different modalities through different branches, capturing key information in feature maps to a greater extent. They also utilize frequency-domain enhancement techniques to obtain global frequency distribution features.
- This study also introduces a space-to-depth downsampling module (SCDown). This module reconstructs features from the spatial dimension to the channel dimension. This process achieves the downsampling split of image channel information. The module then selects key information channels via learnable weights. The transformation reduces the image resolution. It completely retains the key spatial information within the channel dimension. This effectively mitigates the key information loss problem in small object detection.

The remainder of this paper is organized as follows. Section 2 introduces multi-modal object detection models. Section 3 elaborates on the proposed method in detail, including the overall architecture and the design of the ResFuse, DualAtt, HFAtt, and SCDown modules. Section 4 presents the experimental results, including the dataset introduction, implementation details, ablation studies, and comparative experiment analysis. Section 5 and Section 6 provide the discussion and conclusions.

## 2. Related Work

Compared with single-modality detection, multi-modal detection achieves complementary advantages by fusing multi-scale features. RGB extracts high-resolution texture features, and IR supplements all-weather infrared features. Single-modality detection easily fails under severe weather, while multi-modality methods achieve stable detection in all-weather and day-and-night conditions. In this section, we provide an overview of multi-modal object detection models. Specifically, Section 2.1 elaborates on the theoretical model of ICAF multi-modal object detection, and Section 2.2 analyzes the challenges faced by this model in multi-modal object recognition under complex autonomous driving environments.

### 2.1. ICAFusion

**Figure 1:**
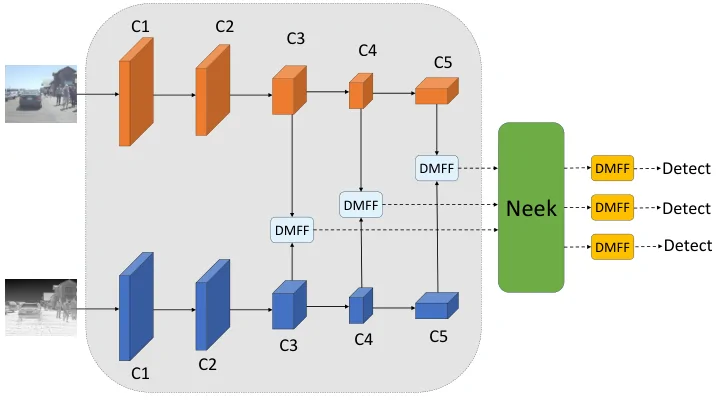
Framework of the multi-modal object detection system.

The framework of the ICAF multi-modal object detection model [23] is shown in Figure 1. First, a dual-branch network extracts features from RGB and IR images. This process can be expressed as in Equation (1):

(1) $$F_{R}^{i}=\Psi_{backbone}\left(I_{R};\theta_{R}\right),F_{T}^{i}=\Psi_{backbone}\left(I_{T};\theta_{T}\right)\;$$

Here, $F_{R}^{i},F_{T}^{i}\in R^{W\times H\times C}$ represent the feature maps from the RGB branch and the IR branch at the *i*-th layer (i=3,4,5), respectively. *W*, *H*, and *C* in $R^{W\times H\times C}$ represent the width, height, and number of channels of the feature map, respectively. $I_{R}$ and $I_{T}$ denote the input RGB image and IR image. $\Psi_{\mathrm{backbone}}$ represents the feature extraction function for the RGB and IR branches with parameters $\theta_{R}$ and $\theta_{T}$.

The feature maps $F_{R}^{i}$ and $F_{T}^{i}$ require cross-modal feature fusion to aggregate features from different branches, which is expressed as in Equation (2):

(2) $$F_{R+T}^{i}=\Phi_{fusion}\left(F_{R}^{i};F_{T}^{i};\theta_{f}\right)$$

Here, $F_{R+T}^{i}\in R^{W\times H\times C}$ represents the fused features in the *i*-th layer. $\Phi_{\mathrm{fusion}}$ denotes the feature fusion function with parameters $\theta_{f}$.

The feature maps ${\left\{F_{R+T}^{i}\right\}}_{i=1}^{L}$ are sequentially fed into the neck network and detector head for classification and regression, which is shown in Equation (3):

(3) $$\left[D_{\mathrm{cls}},D_{\mathrm{bbox}}\right]=\phi_{\mathrm{head}}\left(\phi_{\mathrm{neck}}\left({\left\{F_{R+T}^{i}\right\}}_{i=1}^{L}\right);\theta_{h}\right)$$

Functions $\phi_{neck}$ and $\phi_{head}$ are parameterized with $\theta_{h}$ for classification and bounding box regression. $D_{\mathrm{cls}}$ and $D_{\mathrm{bbox}}$ represent the final output detection boxes and confidences, respectively.

The DMFF module of this model adopts an iterative cross-attention strategy for parameter-sharing computations, while it establishes modal interaction and reduces the spatial complexity. The actual deployment frame rate in autonomous driving scenarios is low because the DMFF module contains parameters from an excessive number of Transformer modules. Therefore, this model must be modified to increase the detection accuracy.

### 2.2. Challenges

(1) The computational load and parameter volume required for directly stacking multiple Transformer modules in this model are massive. Therefore, it is necessary to further optimize the model because edge devices cannot provide sufficient computing power in actual deployment scenarios.
(2) The backbone network of this model neither extracts features at high resolutions nor performs feature fusion, indicating that the contextual correlation of the images needs improvement.
(3) The background information in the images is gradually amplified because excessive interaction between different modal features in the ICFE mechanism produces negative effects with increasing iterations (as shown in Table 1).

## 3. Proposed Method

### 3.1. Design Motivation

The proposed model introduces the following modifications to address the limitations described above:

(1) The design adopts a lightweight mid-level fusion method. This strategy avoids the computational and parameter surge from stacked Transformer modules.
(2) The proposed architecture incorporates different feature extraction modules into the dual-backbone branches. This design enhances contextual correlation for subsequent feature fusion.
(3) The proposed approach further enhances the contrast of the original information within the mid-level fusion process. This strategy suppresses non-essential features during critical information extraction.

Section 3.2 below corresponds to the overall framework of MHF-Net. Section 3.3 solves the different extraction requirements of the dual branches. Section 3.4 adopts a lightweight mid-level fusion method. Section 3.5 further enhances the contextual relationships between images at the feature pyramid level.

### 3.2. Architecture

**Figure 2:**
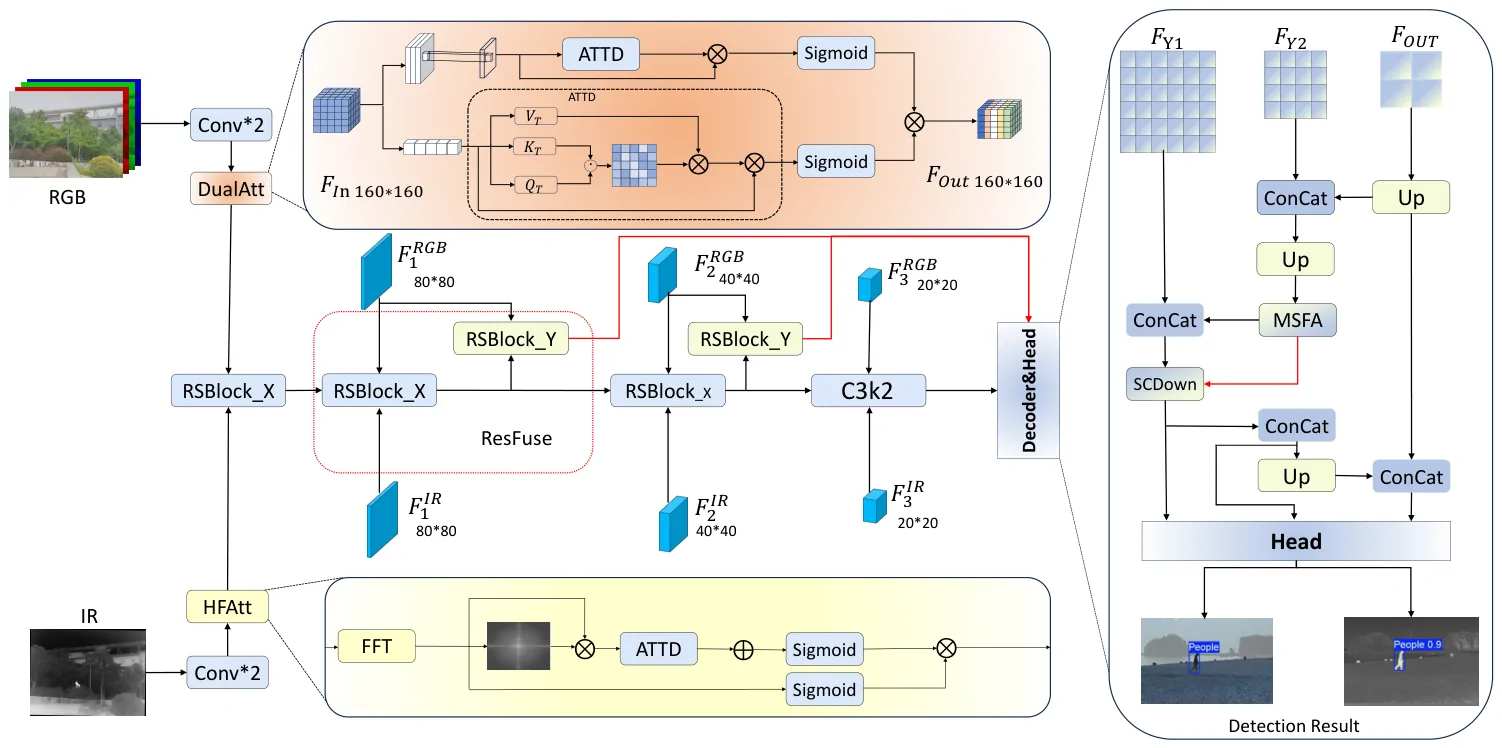
Overall network architecture of MHF-Net.

This paper proposes a multi-modal object detection algorithm to address the excessive computational load and parameter volume while improving the detection accuracy. Figure 2 shows the overall network architecture. This network consists of a dual-branch backbone network, mid-level fusion residual blocks, and a detection head.

First, the dual-branch backbone network processes RGB and thermal images using dual-path attention and high-frequency gated convolution, respectively.

Specifically, the Conv*2 layers use convolutions with a kernel size of 2 and stride of 2. Inside the DualAtt module, the network applies two independent Sigmoid activation functions before the final addition. This process extracts key pixels from each image layer, and it provides sufficient information for the subsequent global context. This can be expressed as in Equations (4) and (5):

(4) $$Z_{R,Ir}^{i}=\Phi_{module}\left(H_{R,Ir}^{i};\omega_{module}\right)$$

(5) $$F_{i}^{RGB,IR}=\Psi_{backbone}\left(Z_{R,Ir}^{i};\theta_{backbone}^{i}\right)$$

Here, $H_{R,\mathrm{Ir}}^{i}\in R^{W\times H\times C}$ represents the input images for dual-path attention processing and high-frequency gated convolution processing, respectively. $F_{i}^{\mathrm{RGB}}$ and $F_{i}^{\mathrm{IR}}$ represent the *i*-th features of the RGB and IR images (i=1,2,3). *H*, *W*, and *C* represent the height, width, and number of channels of the corresponding feature map, respectively. $\Phi_{\mathrm{module}}$ and $\Psi_{backbone}$ represent the basic feature extraction network and feature extraction function for RGB and thermal imaging with parameters $\theta_{backbone}^{i}$ and $\omega_{module}$. In general object detection, VGG16 [24], ResNet [25], and CSPDarkNet [26] are commonly employed as the function $\Psi_{backbone}$. During the feature extraction stage, multi-scale features are typically utilized to capture objects of varying sizes.

Secondly, given that $F_{i}^{RGB,IR}$ represents the feature map input for mid-level fusion, cross-modal feature fusion is necessary to aggregate features from the different branches of the multi-modal object detection network. Cross-modal feature interaction is realized through a multi-modal residual fusion module. Progressive downsampling is employed to align features at various scales, which relaxes the requirement for specific input image sizes. Concurrently, cumulative residuals are utilized to preserve information from preceding stages, thereby improving the efficiency of feature fusion. While maintaining the independent feature extraction capabilities of both modalities, comprehensive information interaction from the bottom to the top layers is achieved. This process can be defined as in Equation (6):

(6) $$F_{R+T}^{i}=\Phi_{fusion}\left(F_{i}^{RGB,IR};\theta_{f}\right)$$

Here, $H_{R,\mathrm{Ir}}^{i}\in R^{W\times H\times C}$ represents the fused features in the i-th layer. $\Phi_{module}$ represents the feature fusion function of mid-level fusion with parameters $\theta_{f}$. Previous studies [27] have explored different fusion architectures. They have verified that intermediate fusion is superior to other fusion methods. Therefore, intermediate fusion is utilized as the default setting to fuse the $F_{i}^{RGB,IR}$ image features from the backbone network, which is shown in Figure 2. This paper proposes a residual block fusion method to simulate $\Phi_{fusion}$. Section 3.4 describes this module in detail.

Finally, the feature maps from $F_{R+T}^{i}$ are input into the detector neck to achieve multi-scale feature fusion. Then, they are passed to the detector head for subsequent classification and regression, which is illustrated in Equation (3):

Here, $\Phi_{head}$ and $\Phi_{neck}$ represent the multi-scale feature aggregation and detection head functions. FPN [28] and PANet [29] usually use the function $\Phi_{neck}$ to enhance the semantic expression and localization capabilities of features.

The “up” operation enlarges the original image size. It uses the nearest neighbor interpolation method.

As shown in Figure 2, the ResFuse module used in this paper adopts a dual-module method for further residual fusion after it extracts features from different branches. The detection head part adopts the structure shown in Figure 2 to perform further fusion on the acquired feature maps, while it uses residual connections to add features.

### 3.3. DualAtt and HFAtt

Image sensors input RGB and IR images. Traditional dual-branch object detection does not process single modalities specifically. Consequently, the network underutilizes the rich target information in high-resolution feature maps. To address this limitation, the proposed architecture adopts the feature extraction scheme shown in Figure 2. The dual attention (DualAtt) and high-frequency gated convolution attention (HFAtt) mechanisms extract edge features in RGB and IR image processing, respectively.

Regarding the dual attention (DualAtt) mechanism, existing traditional cross-attention fusion methods merely perform attention selection on input images, which increases the computational cost and produces limited improvements during training. However, DualAtt selectively extracts the most representative features from the spatial and channel dimensions. This channel and spatial information helps the model to learn critical global and local features more effectively.

Regarding the high-frequency gated convolution (HFAtt) mechanism, existing FFT-based attention modules usually perform global frequency transformations, which destroy the local spatial structures in the features. Furthermore, traditional high-frequency filters easily amplify background noise when they process images under adverse weather conditions. However, HFAtt applies frequency-domain processing to key regions before it combines with a spatial and channel gating mechanism, which highlights fine-grained targets and sharp edge information.

Compared with traditional multi-modal fusion, our method does not simply apply convolution to input features. Instead, it divides the image into two branches, where $F_{D},F_{U}\in R^{W\times H\times C}$, so that it can apply different processing strategies to different branches. Because each branch focuses on different feature points, the model can learn the key information in the feature maps to a greater extent. Then, after the feature maps are input into the ATTD module, the output feature maps are fused with the original feature maps. Finally, the feature maps of the upper and lower branches are added together before they are output.

The input feature maps $F_{D},F_{U}$ are projected into two independent matrices $T_{T},\;T_{R}\in R^{HW\times C}$ so that the network can calculate a set of values and keys; see Equation (7). Then, the tokens of the RGB modality $T_{R}$ are projected into another independent matrix $Q_{R}\in R^{HW\times C}$ so that the network can calculate a set of queries, as shown in Equation (7).

(7) $$V_{T}=T_{T}W^{V},K_{T}=T_{T}W^{K},Q_{R}=T_{R}W^{Q}$$

(8) $$Z_{T}=\mathrm{softmax}\left(\frac{Q_{R}K_{T}^{T}}{\sqrt{D_{K}}}\right)\cdot V_{T}$$

(9) $$F_{i\mathrm{out}}^{i}=\phi_{can}\left(F_{i},Z_{T}\right)$$

(10) $$F_{\mathrm{out}}=\phi_{can}\left(F_{D\mathrm{out}},F_{U\mathrm{out}}\right)$$

Here, $W^{V}$, $W^{K}$, and $W^{Q}$ represent weight matrices. The network constructs the correlation feature matrix through dot product operations. Then, the network performs a normalization operation using the softmax function. The obtained result represents the correlation between RGB image features and IR image features. Finally, the result is multiplied by $V_{T}$ Equation (8) so that the network obtains the vector $Z_{T}$. $F_{i}$ and $F_{\mathrm{iout}}^{i}$ represent the inputs $\left(F_{D},F_{U}\right)$ and outputs of the two branches. Then, the residual fusion function $\phi_{can}$ concatenates the obtained feature matrices so that the network obtains the $F_{\mathrm{iout}}^{i}$ and $F_{out}$ matrices; see Equations (9) and (10).

### 3.4. ResFuse

The network obtains RGB and IR images containing detection target features through the dual attention (DualAtt) and high-frequency gated convolution (HFAtt) mechanisms. Traditional multi-modal fusion cannot fully mine and utilize the salient features of the images if it adopts a direct concatenation method for feature fusion. Therefore, the ResFuse model introduces the residual fusion idea [26].

It adds a residual connection on top of the original feature concatenation. Then, it passes the feature output of the $RSBlock_{X}$ branch to the next level for cascaded residual fusion. Meanwhile, it inputs the features of the $RSBlock_{Y}$ branch directly into the feature pyramid network (FPN).

**Figure 3:**
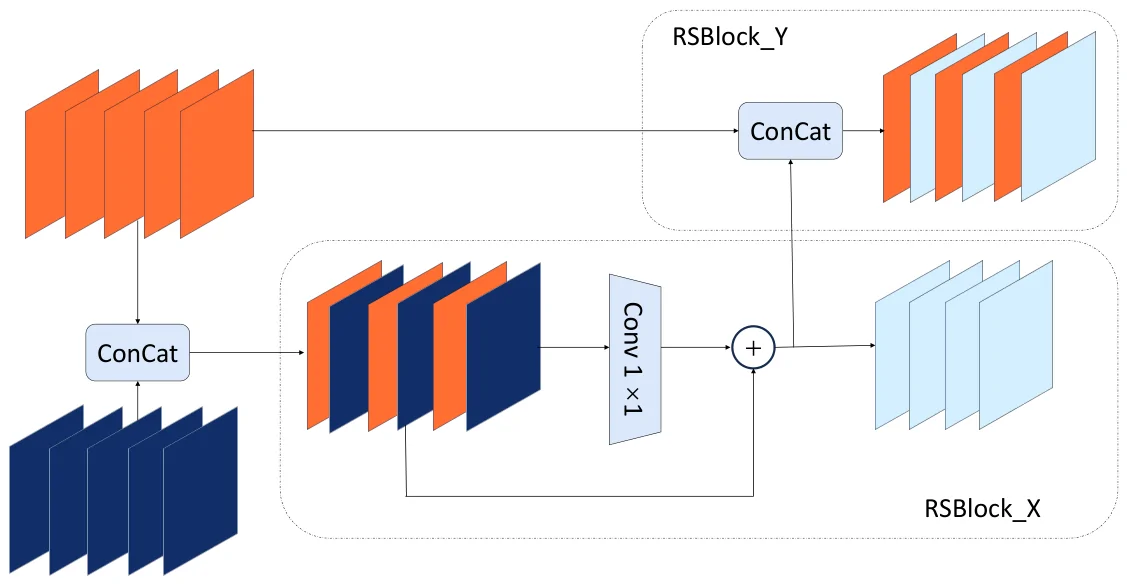
Structure of the ResFuse module.

This module further enhances the features and context relationships of the detection targets by superimposing effective information from high-resolution feature maps based on a small number of parameters. Figure 3 specifically shows the structure of the proposed residual fusion block (ResFuse). It is mainly divided into two modules: $RSBlock_{X}$ and $RSBlock_{Y}$. This method differs from traditional methods of fusing features of different modalities. Residual connections provide a direct path for gradients. This path alleviates the difficulty in training deep networks. Previous-level features directly participate in subsequent calculations so that the network avoids information bottlenecks. The $RSBlock_{Y}$ in the proposed ResFuse retrieves the complementary relationship between RGB and thermal imaging modalities. It also further enhances the pixel values of target features and reduces the pixel values of irrelevant features.

Traditional modality concatenation usually uses the “Concat” operation so that it directly adds channel numbers. Compared to this method, the ResFuse module follows an iterative superposition process. Specifically, the network uses the result of the first concatenation as input for the second concatenation. At the same time, the module performs residual addition based on the first concatenation. This design is highly effective because it not only retains the initial key pixel points but also provides edge weights for subsequent feature extraction.

Given two input feature maps $F_{R},F_{Ir}\in R^{W\times H\times C}$, the proposed method first concatenates the two feature maps along the channel dimension. The feature maps form a tensor in $R^{W\times H\times 2C}$, denoted as $F_{RIr\_1}$. Following feature extraction, it is concatenated again in 2D with $F_{R}$ to obtain $F_{RIr\_2}$. $F_{RIr\_1}$ is fed into the residual connection block of the subsequent layer, while $F_{RIr\_2}$ is forwarded to the detection head. For the sake of clarity, this is expressed in Equation (11) below:

(11) $$F_{\left\{RIr\_x\right\}}^{i}=\left\{\begin{array}{cc} \varphi_{\left\{can\right\}}\left(F_{R}^{t},F_{\left\{Ir\right\}}^{t}\right), & t=1\\ \varphi_{\left\{can\right\}}\left(F_{\left\{RIr\right\}}^{t},F_{\left\{Ir\right\}}^{t},F_{R}^{t}\right), & t>1 \end{array}\right.$$

Here, t represents the n-th time of multi-modal residual fusion $\left(n=1,\;2,\;3\right).\;F_{\mathrm{RIr}}^{i}$ is the output fused feature map. We set i=t=1,2,3. Meanwhile, x has two conditions. The network sends the map to the subsequent residual fusion module when x = 1, while the network sends the map to the detection head for feature fusion when x=2. The equivalent formulas are as follows:

(12) $$F_{RIr}=F_{RIr\_1}$$

(13) $$F_{N}=\Phi_{Net}\left(F_{RIr\_2}^{i},F_{-1}\right)$$

Here, $\Phi_{Net}$ is the inference function of the subsequent detection head. $F_{-1}$ represents the feature map output by the previous layer. The equation $F_{RIr}=F_{RIr\_1}$ ensures the input for the subsequent residual module.

### 3.5. SCDown and MSFA

The network obtains an image containing texture features of the RGB image and edge features of the IR image after multi-modal residual fusion (ResFuse). Then, the network needs to input the image into the feature pyramid. Table 2 shows the traditional downsampling operation. Strided convolution and pooling inevitably cause information loss. This is particularly disadvantageous given the image blurring problem caused by poor weather. The network needs to further enhance the context relationships when it inputs the multi-modal fusion and dual-branch feature maps into the feature pyramid network (FPN). Conventional downsampling may completely eliminate their representations because small targets occupy only limited pixels in the feature map.

Traditional max pooling directly discards 75% of the window information. Strided convolution destroys the spatial coherence of features. This operation also incurs a large computational overhead. The spatial-channel downsampling (SCDown) module divides the channels into four parts. Learnable weights determine the retention of key parts. This mechanism effectively extracts important small targets in images with excessive background noise.

Regarding traditional multi-scale feature fusion modules, inputting images directly into convolutional layers for feature extraction leads to high computational costs. However, our multi-scale feature aggregation (MSFA) module adopts a combination strategy of “split, partial processing, and fusion”. This strategy only slightly increases the number of parameters. At the same time, it preserves the initial edge information and transmits it directly to the next layer.

**Figure 4:**
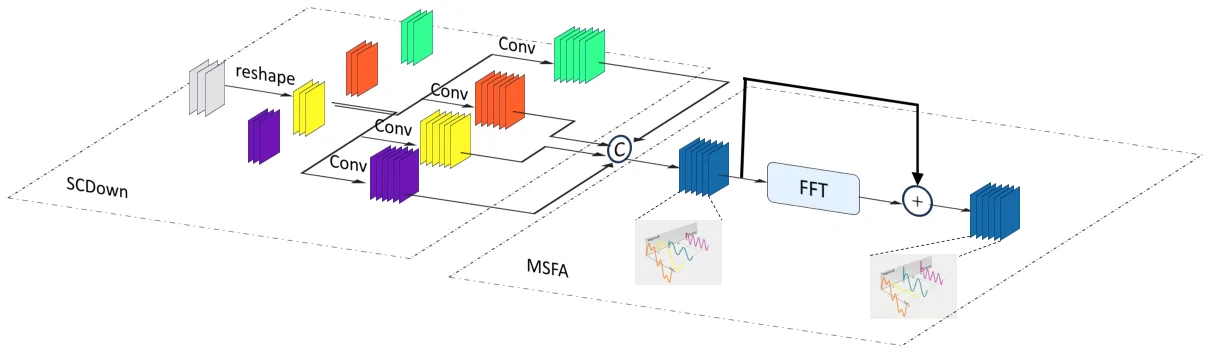
Block diagram of the SCDown module structure.

For example, its representation may only have 1 pixel left or even disappear completely after a 4×4 target undergoes two 2×2 pooling operations. This paper proposes the space-to-depth convolution (SCDown) module to solve this problem. Figure 4 shows its spatial structure diagram.

The SCDown module implements a space-to-channel transformation. Given an input feature map $X\in R^{B\times C\times H\times W}$, the space-to-depth operation divides the spatial dimension into 2 × 2 sub-regions. Then, it reorganizes them along the channel dimension. Its mathematical expression is given in Equation (14):

(14) $$X_{SPD}=\mathrm{Concat}\left(X_{::2,::2},X_{1::2,::2},X_{::2,1::2},X_{1::2,1::2}\right)$$

Here, $X_{::2,::2},X_{1::2,::2},X_{::2,1::2}$, and $X_{1::2,1::2}$ represent the top-left, top-right, bottom-left, and bottom-right positions in each 2×2 region, respectively. $X_{SPD}$ represents the output feature matrix.

In summary, Table 3 shows the number of parameters and the computational complexity of the modules used in this paper. The model maintains the FPS while it reduces the computational complexity and the number of parameters. This is beneficial for object detection under the image blurring problem caused by poor weather.

## 4. Experiments

### 4.1. Dataset

Jinyuan Liu et al. established the M3FD dataset [12]. A synchronized system captured this dataset in different scenarios. The system consisted of vehicle-mounted binocular optical cameras and binocular infrared sensors. This public dataset reflects dual visible–thermal infrared modalities. It contains 34,407 manually annotated labels. These labels adopt the VOC format. The dataset includes six target categories: person, car, bus, motorcycle, lamp, and truck. The dataset is partitioned into 2940 training images, 630 validation images, and 630 test images.

This study evaluates various performance aspects of MHF-Net through comparative and ablation experiments on the M3FD dataset [12]. The evaluation verifies the effectiveness and robustness of the algorithm. The main scenarios include the State Tourism Holiday Resort at the Golden Stone Beach in Dalian, China; the main roads in Jinzhou District Dalian, China; and the Campus of Dalian University of Technology. The total number of images is 8400. The dataset contains 600 independent scenes. The image size is 1024 × 768 pixels.

### 4.2. Experimental Setup

Our method was implemented using the PyTorch 2.7.1 framework on a Windows server equipped with an Intel Xeon Gold 6248R CPU, 64 GB of RAM, and an NVIDIA RTX 6000 Ada GPU (44 GB). The models were trained for 500 epochs with a batch size of 16. The network was optimized using stochastic gradient descent (SGD) with an initial learning rate of 0.01, momentum of 0.937, and weight decay of 0.0005. A cosine annealing schedule was applied for learning rate decay. For both training and testing, the input image resolution was set to 640 × 640 pixels. Additionally, data augmentation techniques, including mosaicking and random flipping, were employed during training.

### 4.3. Evaluation Metrics

This study employs standard quantitative metrics to evaluate model performance. Specifically, the precision (P), recall (R), average Precision (AP), mean average precision (mAP), F1 score, frames Per second (FPS), and model size were adopted. Their respective formulas are given in the following.

Precision is the percentage of correctly predicted positive samples out of all samples predicted as positive. The calculation formula for precision is as follows:

(15) $$P=\frac{\mathrm{TP}}{\mathrm{TP}+\mathrm{FP}}$$

Here, P is precision; TP is the number of correctly predicted positive samples; and FP is the number of negative samples incorrectly predicted as positive. Recall is the percentage of correctly predicted positive samples out of the actual positive samples. The calculation formula for recall is as follows:

(16) $$R=\frac{\mathrm{TP}}{\mathrm{TP}+\mathrm{FN}}$$

FN is the number of positive samples incorrectly predicted as negative, and R is recall.

(17) $$F1=2\times \frac{P\times R}{P+R}$$

The mean average precision (mAP) is utilized to evaluate the overall detection accuracy across all categories. It is defined as the mean of the average precision (AP) for each class *i* over *N* total categories, where AP represents the area under the precision–recall (P-R) curve. The equations are as follows:

(18) $$\mathrm{AP}=\int_{0}^{1}P\left(R\right)dR$$

(19) $$\mathrm{mAP}=\frac{1}{N}\sum_{i=1}^{N}{\mathrm{AP}}_{i}$$

### 4.4. Ablation Study

This study further quantifies the independent contributions of each module. It also verifies their synergistic effects. The evaluation uses the same dataset, training, and backbone settings. This research includes combinatorial validation on ResFuse, DualAtt, HFAtt, and SCDown and MSFA. Table 4 shows the details. Certain experimental configurations omit the corresponding innovative modules. These configurations replace the original modules with C3K2 and C2PSA. This replacement ensures comparability by restoring the baseline model.

As shown in Table 4, in terms of overall results, the complete MHF-Net achieves precision of 87.4%, an F1 score of 82.9%, recall of 79.2%, an mAP_50_ of 84.3%, and an mAP_50_90_ of 57.6%. Compared to the baseline model lacking these four modules, these five metrics are improved by 2.9%, 3.6%, 4.9%, 4.3%, and 4.0%, respectively. This demonstrates that the integration of these three modules yields significant improvements in the multi-modal object detection model.

Furthermore, the single-module comparison reveals that the ResFuse module alone achieves an mAP_50_ of 81.2% and an mAP_50_90_ of 54.7%, improving upon the baseline model by 1.2% in the mAP_50_. The precision also rises to 85.5%, indicating that multi-level residual fusion facilitates key information extraction and enhances the model’s judgment. Meanwhile, enabling DualAtt yields precision of 87.7% and recall of 76.2%. The combination of DualAtt and HFAtt further improves the mAP_50_ and mAP_50_90_ by 1.2% and 0.5%, respectively, compared to DualAtt alone, reaching an mAP_50_ of 82.8%. This demonstrates that the HFAtt module effectively improves edge sharpness in IR images and suppresses bad-weather noise. Similarly, enabling only SCDown and MSFA yields an mAP_50_ of 83.1%. Both configurations outperform the baseline, providing stable performance gains.

Evaluating partial module combinations, the combination of ResFuse, DualAtt, and HFAtt yields 82.3% for the mAP_50_ and 56.3% for the mAP_50_90_. Compared to the standalone use of SCDown and MSFA, these values decrease by 0.8% and 1.3%, respectively. This suggests that, without fused feature aggregation, frequency-domain and global residuals have an upper limit in compensating for multi-modal fusion boundary shaping. Furthermore, when comparing the combination of ResFuse and SCDown and MSFA (83.4% for mAP_50_, 57.2% for mAP_50_90_) with SCDown and MSFA alone, the addition of ResFuse marginally increases the mAP_50_ by 0.3% and recall by 1.1%, despite the slight decreases in precision (0.1%) and the mAP_50_90_ (0.4%). This increased recall indicates an advantage in image classification accuracy. Comparing the combination of ResFuse and SCDown and MSFA against the combination of DualAtt, HFAtt, and SCDown and MSFA reveals increases in the mAP_50_, mAP_50_90_, and recall, but a decrease in precision. These performance fluctuations demonstrate that incomplete modular synergy disrupts the feature balance.

Specifically, while ResFuse explicitly sharpens the edges of the fused image, adding SCDown and MSFA (global self-attention) without the frequency-domain noise filtering of DualAtt and HFAtt causes the global attention mechanism to inadvertently model irrelevant background noise (e.g., trunk edges or railing boundaries) alongside the target. This inadvertently dilutes the sharpened boundaries. Conversely, omitting the global semantic constraints causes the high-frequency enhancement of DualAtt and HFAtt to overamplify local non-target objects (e.g., a person wearing a raincoat riding an electric scooter), causing misclassification and reducing the precision.

Ultimately, the system achieves a closed-loop balance through the complete synergy of all four modules, peaking at an mAP_50_ of 84.3% and an F1 score of 82.9%. In this configuration, ResFuse anchors the structural boundaries, DualAtt and HFAtt filter frequency-domain noise, and SCDown and MSFA ensure cross-regional semantic consistency, fully mitigating the negative impacts of the partial modules. As verified by our visualization results, this synergy increases the mAP_50_ to 84.3% beyond the highest-performing partial combination, reflecting the vital complementarity of the four modules in multi-modal feature fusion.

### 4.5. Comparison with Mainstream Models

We selected YOLOv8s [30], YOLOv10s [4], YOLOv11s [31], RT-DETR-R18 [3], ICAF [23], SuperYOLO [32], CMA-DET [33], and CFT [34] as comparison models so that we could systematically evaluate the overall detection capabilities and cross-category generalization ability of MHF-Net and provide an objective reference standard.

To ensure fairness, we conducted comprehensive control experiments under a unified data partition, input scale, and training strategy. Specifically, we used a unified SGD optimizer and the batch size was set to 16. Single-modality networks do not use modality fusion. For multi-modal networks, the modality input protocol uniformly adopted the “middle fusion” method. Furthermore, the comparative networks used the default training epochs provided by their original codes.

**Figure 5:**
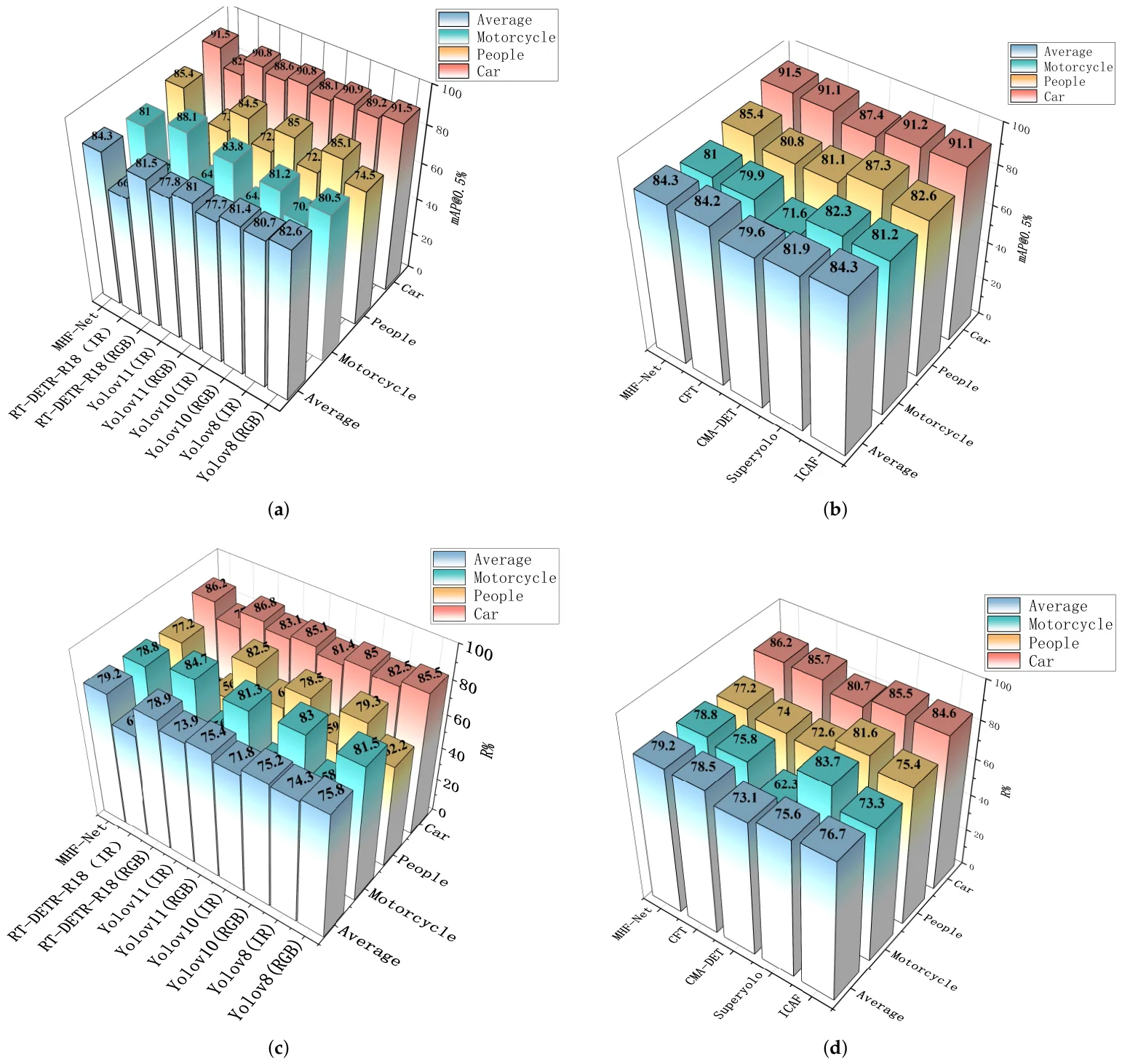
(**a**) Histogram of mAP_50_ for single-modality comparison. (**b**) Histogram of mAP_50_ for multi-modality comparison. (**c**) Histogram of R% for single-modality comparison. (**d**) Histogram of R% for multi-modality comparison.

The study conducted comprehensive controlled experiments under a unified data partition, input scale, and training strategy. The evaluation considers the performance across the four categories and the overall performance. Precision, recall, the F1 score, and the mAP_50_ serve as the evaluation metrics. The training process was repeated five times. The evaluation averages the final results. Table 5 and Figure 5 detail the average experimental results.

As shown in Table 4 and Table 5, MHF-Net achieves an mAP_50_ of 84.3%, an F1 score of 82.9%, precision of 87.4%, and recall of 79.2%. These metrics demonstrate its highly competitive performance among all evaluated methods, highlighting the proposed architecture’s ability to effectively balance detection accuracy and inference speed.

**Figure 6:**
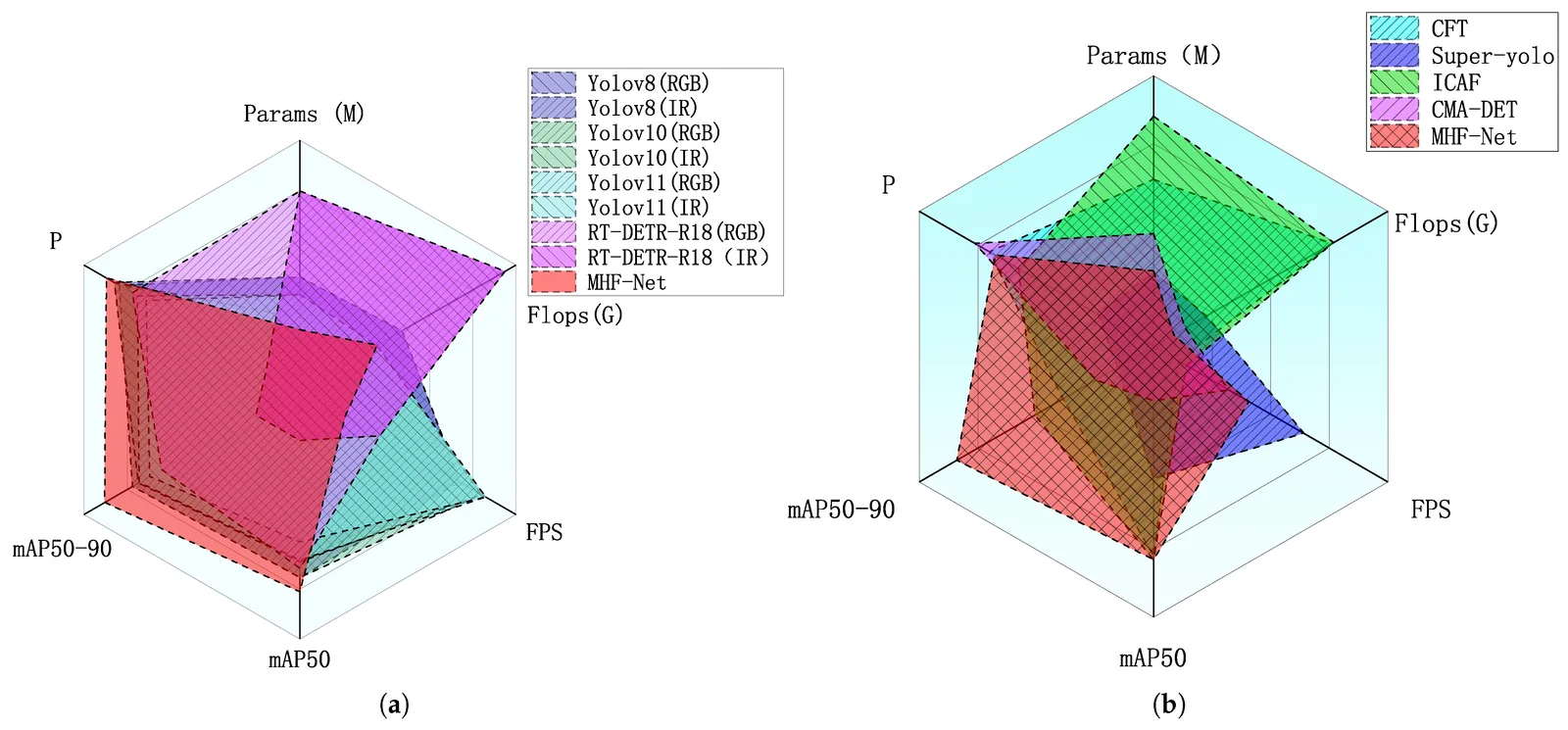
(**a**) Radar chart for single-modality comparison. (**b**) Radar chart for multi-modality comparison.

These results comprehensively demonstrate that MHF-Net simultaneously improves the accuracy and robustness in the evaluated scenarios. To more intuitively present the comparative results and category trends from Table 5, Figure 6 is included to display the parameters (M), FLOPs (G), FPS, mAP_50_, mAP_50_90_, precision (P), and recall (R) for each evaluated model.

Compared to the existing object detection models listed in Table 5, MHF-Net demonstrates significant performance advantages across most evaluation metrics. Specifically, MHF-Net achieves an mAP_50_ of 84.3%, an mAP_50_90_ of 57.6%, precision of 87.4%, and recall of 79.2%, substantially outperforming the baseline models. For instance, the single-modal YOLO11 only achieves an mAP_50_ of 80.5% (precision 83.8%, recall 75.4%) on RGB inputs and an mAP_50_ of 78.3% (precision 82.8%, recall 73.9%) on IR inputs.

When evaluating multi-modal (RGB + IR) architectures, SuperYOLO achieves an mAP_50_ of 81.9% and precision of 83.9%. Furthermore, while advanced models like ICAF reach a competitive mAP_50_ of 84.3% (precision 86.6%, recall 76.7%) and CFT attains an mAP_50_ of 84.2% (precision 87.6%, recall 78.5%), MHF-Net still maintains overall superiority. Most notably, MHF-Net dominates in the more stringent mAP_50_90_ metric (57.6%), further highlighting its robust detection capabilities.

In addition to its outstanding accuracy, MHF-Net achieves a real-time processing speed of 61 FPS, significantly surpassing other high-accuracy multi-modal models such as CFT (16 FPS) and ICAF (21 FPS). Among the evaluated multi-modal models, while SuperYOLO attains the highest inference speed at 96 FPS, it suffers from a substantial drop in detection performance, achieving lower precision of 83.9%, an mAP_50_ of 81.9%, and a significantly degraded mAP_50_90_ of 47.3%. These quantitative results comprehensively demonstrate the practical applicability of MHF-Net: it maintains a favorable balance between high detection accuracy and efficient real-time processing, making it highly suitable for potential deployment in real-world surveillance and autonomous driving scenarios.

The comparative experimental results demonstrate that MHF-Net effectively improves the detection capabilities in autonomous driving environments. Specifically, the model achieves precision of 87.4%, recall of 79.2%, and an mAP_50_ of 84.3%. Compared to the baseline model, these precision, recall, and mAP_50_ values represent significant increases of 2.9%, 4.9%, and 4.3%, respectively.

Furthermore, regarding model efficiency (Table 5), MHF-Net maintains a highly lightweight architecture with only 6.1M parameters and computational complexity of 21.5 GFLOPs. In contrast, other multi-modal models impose significantly larger computational burdens, including CFT (73.6M, 190.1 GFLOPs), ICAF (120.2M, 192.1 GFLOPs), and CMA-DET (33.4M, 33.79 GFLOPs). Although SuperYOLO features a slightly smaller parameter count (4.8M) than MHF-Net, its mAP_50_ and precision are substantially lower. Therefore, by leveraging an efficient residual fusion structure, MHF-Net achieves an optimal balance among detection precision, model size, and inference speed, making it highly suitable for deployment in general lightweight industrial and autonomous driving scenarios.

### 4.6. Qualitative Analysis

**Figure 7:**
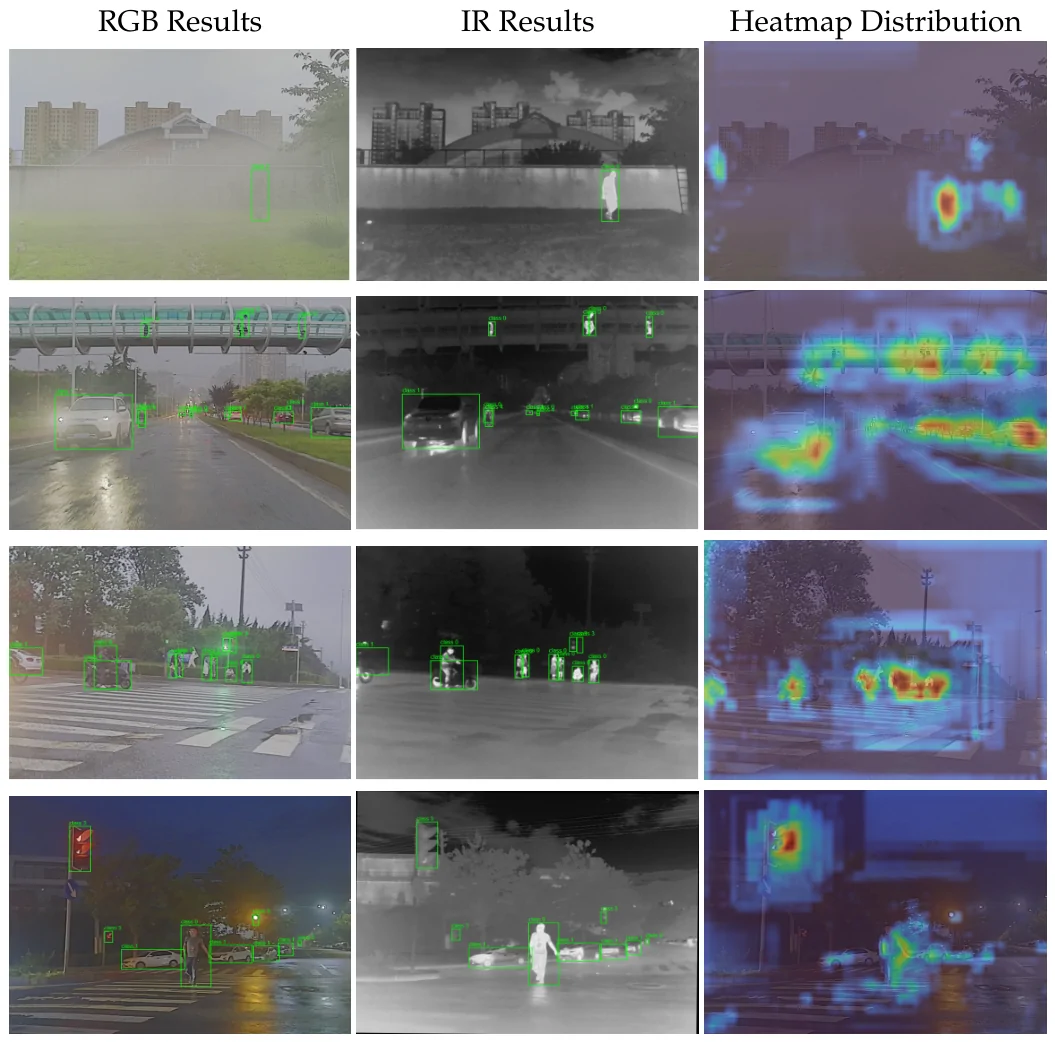
Each row represents a set of inputs, outputs, and heatmaps. The (**left**) column shows the RGB image results. The (**middle**) column shows the IR image results. The (**right**) column shows the heatmap distribution of MHF-Net.

Figure 7 shows the sample visualization results in terms of daytime and nighttime attention maps on the M3FD dataset. Figure 7 illustrates this scenario. The naked eye cannot easily detect pedestrians in RGB images under low-light conditions. Furthermore, other factors in complex urban environments may affect the detection and localization accuracy of pedestrians. Although the constraint of mixed pedestrians, vehicles, and traffic scenes poses a significant challenge, the proposed method can distinguish between different object categories with the help of auxiliary modalities. Our method can capture highly distinguishable features by utilizing global spatial location information and the correlations between different objects, as Figure 7 shows.

### 4.7. Real-World Testing

**Figure 8:**
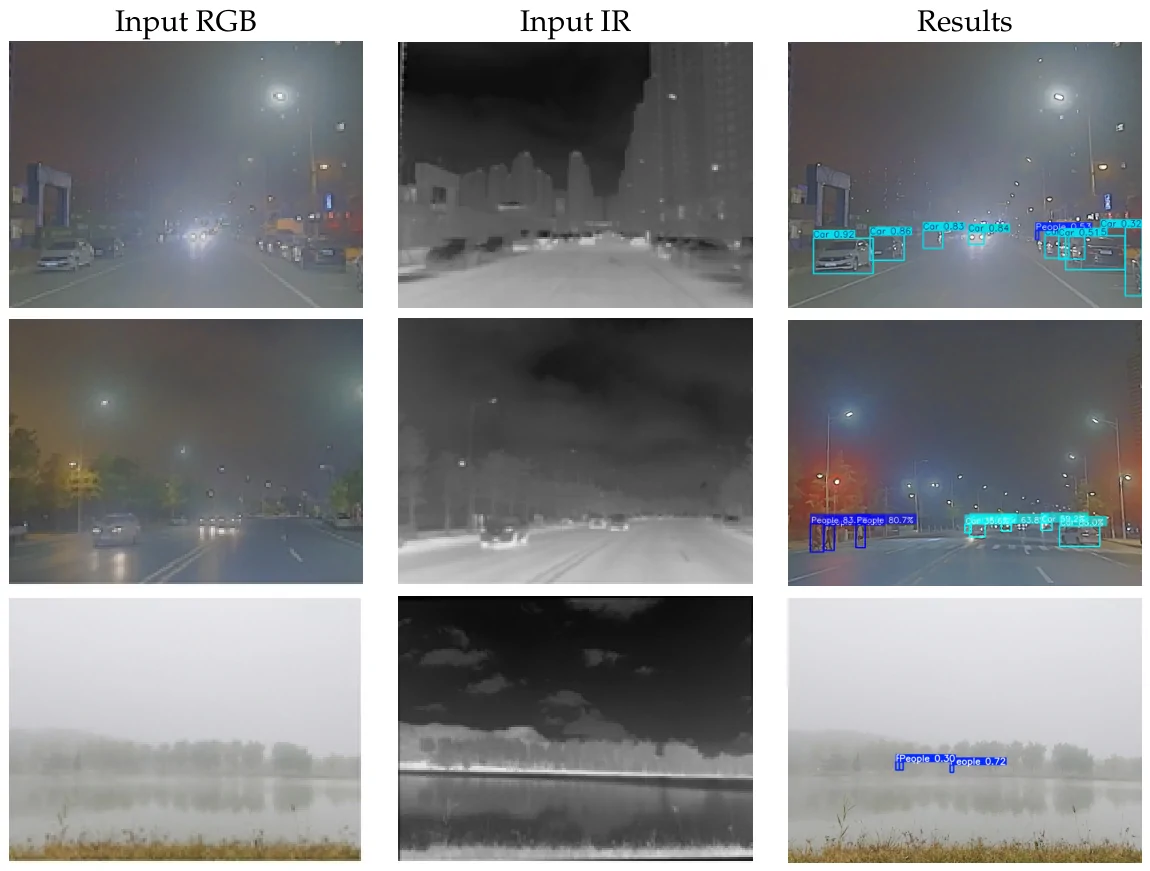
Each row is a set of real captured photos. The (**left**) part shows the RGB images (including rainy, foggy, and overexposed photos) that MHF-Net receives as input. The (**middle**) part shows the IR images that MHF-Net receives as input. The (**right**) part shows the detection results of MHF-Net.

This section qualitatively evaluates the practical applicability of MHF-Net. The data acquisition device consists of a synchronized system. The system includes a binocular optical camera and a binocular infrared sensor. The visualization results demonstrate the practical applicability of the model in real-world scenarios. The resolution of visible light images is 1024×768, and the standard resolution of infrared images is also 1024×768. The data collection scenes are major roads in Jinzhou District, Dalian City. Images were randomly selected from real-world scenarios as input for the network to perform inference and detect corresponding objects. The results are illustrated in Figure 8.

### 4.8. Failure Case Analysis

**Figure 9:**
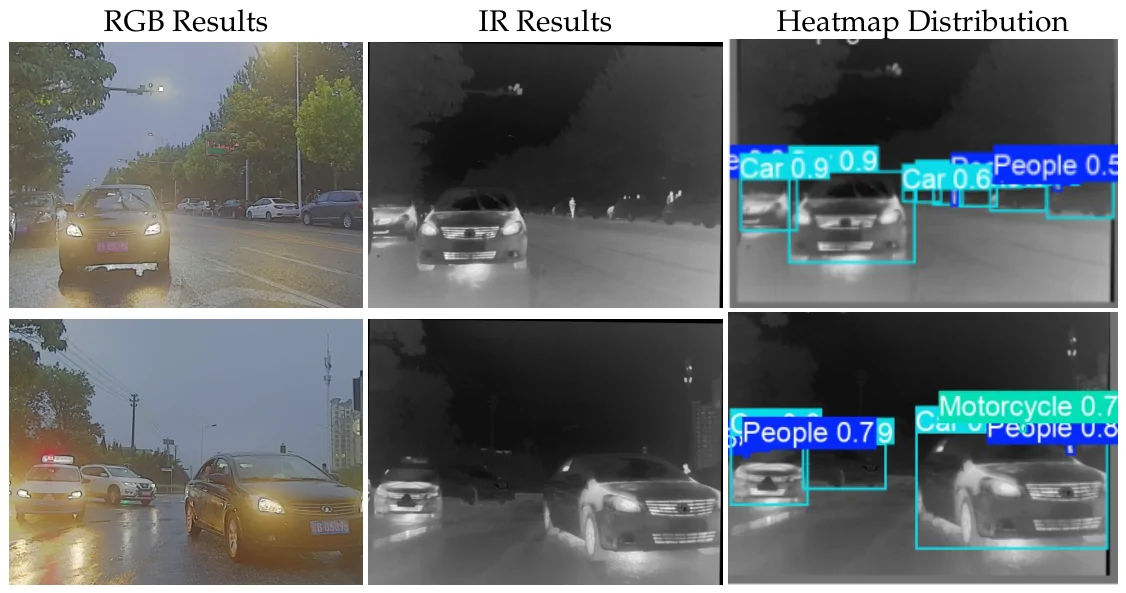
Each row represents a set of RGB, IR, and detection images. The (**left**) column shows the ground truth RGB image. The (**middle**) column shows the ground truth IR image. The (**right**) column shows the detection result of MHF-Net.

Figure 9 shows the recognition errors of the proposed model. These errors occur under unique circumstances. For example, some targets are incomplete. Faces appear inside car windows. Severe interference affects the IR images. Several factors cause these target detection failures. The IR environment severely affects the judgment of RGB images. Furthermore, motorcycles appear similar to cars at close range.

## 5. Discussion

First, a natural class imbalance exists within the dataset, particularly regarding the limited number of lamp (streetlight) samples (i.e., there are only 77 images in the test set). This uneven distribution does not accurately reflect the typical conditions of real-world autonomous driving scenarios. Furthermore, the fusion of feature maps within the multi-modal framework serves as a structural regularizer. This mechanism effectively mitigates the overfitting tendencies typically associated with scarce single-modality RGB data. Nevertheless, continuously expanding the dataset to balance minority classes remains a priority for future work.

Second, despite achieving high overall detection performance, MHF-Net still encounters difficulties in certain challenging scenarios. For instance, when detecting very distant targets, the visual appearances of cyclists and pedestrians can become highly similar. In such cases, the lack of distinct depth and structural variations can occasionally lead to missed detections. Additionally, under conditions where vehicles and pedestrians overlap, severely degraded RGB textures can result in slight deviations in bounding box regression. These failure cases highlight the inherent challenges of real-world optical detection and underscore the necessity of critical evaluation when interpreting visual features.

Ultimately, the modality dimensions of the current framework require further expansion. Future work will explore the incorporation of additional sensory modalities, such as near-infrared imaging, depth sensing, LiDAR, or radar. Integrating these sensors will facilitate the evaluation and improvement of the detection robustness in highly complex field environments.

## 6. Conclusions

To address the limitation whereby most existing multi-modal object detection methods rely on simple feature fusion, this paper proposes a multi-modal residual fusion object detection framework based on high-frequency gated convolutions, aiming to overcome the challenge of image blurring caused by severe weather conditions. Specifically, the framework first effectively enhances the target features in both RGB and IR images through high-frequency gated convolutions and a spatial-channel mechanism. Subsequently, a multi-modal residual fusion module is introduced to optimize the final feature representation by utilizing global complementary information across different modalities, thereby significantly improving the model performance with only a marginal increase in parameters.

By effectively addressing issues caused by severe weather, such as overexposure and partial occlusion, the proposed method demonstrates robust object detection under challenging environmental conditions. Consequently, this capability offers a highly promising framework for smart city applications, including environmental monitoring and autonomous driving. Future work will explore more efficient and lightweight cross-modal feature fusion frameworks.

## Figures and Tables

**Table 1:** Comparison of ICFE across different numbers of iterations.

Number	mAP50	Params (M)	FPS
0	77.5	528.5	40.5
1	79.2	528.5	36.7
2	76.9	528.5	32.8
3	77.5	528.5	29.6

**Table 2:** Comparison of traditional methods.

Method	Operating Principle	Applicable Scenarios	Information Retention
Max Pooling	Extracts the local maximum	General	Loses 75% of information
Average Pooling	Extracts the local average	General	Information blurring
Strided Convolution	Learnable downsampling	General	Partial loss
SCDown	Spatial-to-channel recombination	Small object detection	Completely retained

**Table 3:** Network parameters with superimposed modules.(Here, √ represents the enabled module and × represents the disabled module).

Model	ResFuse	DualAtt	HFAtt	SCDown and MSFA	Params (M)	FLOPs (G)	FPS
MHF-Net	√	√	√	√	6.1	21.5	61
	√	√	√	×	5.5	15.5	64
	√	√	×	×	5.5	14.7	67
	√	×	×	×	5.4	13.2	79

**Table 4:** Ablation study of the proposed network. (Here, √ represents the enabled module and × represents the disabled module).

Model	ResFuse	DualAtt	HFAtt	SCDown and MSFA	P%	F1%	R%	MAP_50_	MAP_50_90_
MHF-NET	√	√	√	√	87.4	82.9	79.2	84.3	57.6
	√	×	×	√	86.4	81.8	78.6	83.4	57.2
	×	√	√	√	87.3	81.4	75.3	82.5	56.4
	√	√	√	×	86.6	82.0	76.9	82.3	56.3
	×	×	×	√	86.5	81.6	77.5	83.1	57.6
	×	√	√	×	88.6	81.3	75.6	82.8	56.3
	×	√	×	×	87.7	81.2	76.2	81.6	55.8
	√	×	×	×	85.5	80.0	76.5	81.2	54.7
	×	×	×	×	84.5	79.3	74.3	80.0	53.6

**Table 5:** Model parameters in the comparative experiments.

Model	Modality	Params (M)	FLOPs (G)	FPS	mAP_50_	mAP_50_90_	P	R
**YOLO8**	**RGB**	11.3	28.4	114	82.6	54.4	85.9	75.8
**YOLO8**	**IR**				80.7	53.7	86.5	74.3
**YOLO10**	**RGB**	7.2	21.4	124	82.6	54.4	85.9	75.2
**YOLO10**	**IR**				80.7	53.7	86.5	71.8
**YOLO11**	**RGB**	9.5	21.3	126	80.5	53.6	83.8	75.4
**YOLO11**	**IR**				78.3	52.4	82.8	73.9
**DETR-R18**	**RGB**	19.9	57.0	77	81.5	51.0	84.4	78.9
**DETR-R18**	**IR**				66.1	40.1	68.9	62.4
**CFT**	**RGB + IR**	73.6	190.1	16	84.2	50.8	87.6	78.5
**SuperYOLO**	**RGB + IR**	4.8	56.6	96	81.9	47.3	83.9	75.6
**ICAF**	**RGB + IR**	120.2	192.1	21	84.3	52.6	86.6	76.7
**CMA-DET**	**RGB + IR**	33.4	33.79	48	79.6	48.7	88.1	73.1
**MHF-Net**	**RGB + IR**	6.1	21.5	61	84.3	57.6	87.4	79.2

## Data Availability

The original data and code presented in the study are openly available in GitHub at https://github.com/dlut-dimt/TarDAL (accessed on 1 September 2026) (DOI: 10.1109/CVPR52688.2022.00571).
